# Retention, savings and interlimb transfer of reactive gait adaptations in humans following unexpected perturbations

**DOI:** 10.1038/s42003-018-0238-9

**Published:** 2018-12-14

**Authors:** Christopher McCrum, Kiros Karamanidis, Paul Willems, Wiebren Zijlstra, Kenneth Meijer

**Affiliations:** 1 Department of Nutrition and Movement Sciences, NUTRIM School of Nutrition and Translational Research in Metabolism, Maastricht University Medical Centre+, P.O. Box 616, Maastricht, 6200 MD The Netherlands; 2 Institute of Movement and Sport Gerontology, German Sport University Cologne, Am Sportpark Müngersdorf 6, Cologne, 50933 Germany; 3 Sport and Exercise Science Research Centre, School of Applied Sciences, London South Bank University, 103 Borough Road, London, SE1 0AA UK

**Keywords:** Motor control, Neurophysiology

## Abstract

Reactive locomotor adaptations are crucial for safe mobility, but remain relatively unexplored. Here we assess reactive gait adaptations, and their retention, savings and interlimb transfer. Using new methods to normalise walking speed and perturbation magnitude, we expose eighteen healthy adults to ten unexpected treadmill belt accelerations during walking (the first and last perturbing the right leg, the others perturbing the left leg) on two days, one month apart. Analysis of the margins of stability using kinematic data reveals that humans reactively adapt gait, improving stability and taking fewer recovery steps, and fully retain these adaptations over time. On re-exposure, retention and savings lead to further improvements in stability. Currently, the role of interlimb transfer is unclear. Our findings show that humans utilise retention and savings in reactive gait adaptations to benefit stability, but that interlimb transfer may not be exclusively responsible for improvements following perturbations to the untrained limb.

## Abstract (web summary)

Christopher McCrum et al. present a study of reactive gait adaptation in humans using unexpected treadmill accelerations during walking. They find that humans improve their gait stability over a short time period and, at re-exposure one month later, show further stability improvements due to retention and savings.

## Introduction

Human locomotion is highly adaptable to environmental change^1–3^, which ensures effective and safe mobility in daily life. Humans are capable of rapidly adapting gait kinematics in both reactive^4–6^ and predictive^3,7^ manners and such adaptation can be retained over time^3,8–10^ and transferred between different locomotor tasks and environmental conditions^11–16^. In particular, reactive gait adaptations are of great interest for falls prevention research and are the focus of perturbation-based balance training for populations at an increased risk of falls^5,10,17–21^. However, the retention (a preservation over time of adaptations made previously), savings (faster re-adaptation on re-exposure to a perturbation) and transfer (changes in an untrained limb or task reflecting, at least to some extent, the changes seen in the trained limb or task) of reactive gait adaptations are not yet well understood, despite their importance for falls prevention interventions. Reactive gait adaptability implies that the neuromuscular system can alter its behaviour in a feedback-driven manner, meaning that modulation of spinal and sensory reflex pathways may be occurring. Although spinal plasticity, in general, is well supported in humans^22,23^ and has been demonstrated during walking in animals^24–28^, little is known about whether specifically reactive gait adaptations in humans are amenable to savings and transfer between the lower limbs. These adaptation qualities may be quite different from those occurring during predictive gait adaptation, which involve supraspinal processes^29,30^.

Two examples of unilateral lower limb reflexes that are purported to support safe locomotion are limb withdrawal reflexes during the stance phase^31^ and stumble correction reflexes during the swing phase^32^ of gait (i.e., quick removal of the limb if an unsafe object is stepped upon during the stance phase or contacted during the swing phase). It has also been suggested that interlimb reflexes (as evidenced by responses in the contralateral limb following perturbation of the ipsilateral limb) support gait stability control^33–37^. Note that these studies have used a variety of methods to perturb the lower limbs, including direct nerve stimulation, single joint perturbations and perturbations that have a whole-body effect, which may result in very different responses and adaptations. Such stumbling and interlimb reflexes have also been observed^38,39^ and have been shown to adapt^38,40^ following repeated simulated trip perturbations in infants prior to independent walking, indicating that adaptation of these reflexes can occur in a feedback-driven manner, without substantial supraspinal influence. That is not to say that supraspinal structures do not influence balance control in human adults, as there is ample evidence to the contrary^36,41–48^, but our knowledge of the supraspinal influence on reactive gait stability control during unexpected mechanical perturbations, specifically, is currently limited.

Despite evidence of feedback-driven adaptation in these reflexes during specific stimulation or joint level perturbations and in gait stability control following whole-body mechanical perturbations, whether or not this translates to the retention, savings and interlimb transfer of adaptations in reactive gait stability following mechanical, whole-body perturbations such as slips and trips has not, to our knowledge been addressed in the literature. There is evidence to suggest that humans can at least partly retain reactive adaptations in gait stability over different time periods of months to years^8–10,49^. However, no study has examined savings and the interlimb transfer of reactive gait adaptations to standardised, controlled whole-body (mechanical) perturbations. As these processes are of both fundamental and clinical relevance for understanding human locomotor control, further research into these processes is warranted.

Here we assess the reactive adaptation of gait in response to unexpected, repeated gait perturbations in young healthy adults, how this adaptation is retained after 1 month, and if savings and interlimb transfer of these adaptations can be observed. To achieve this in as controlled and as precise a manner as possible, we use new methods to decrease inter-individual differences in gait stability via a normalisation of walking speed based on gait stability and by perturbing gait with a treadmill belt acceleration standardised to the stability-normalised walking speed^50^ (preprint version). Thereby, we account for the effects of walking speed on gait stability control and measurement that we have previously outlined^5,50,51^. The margin of stability (MoS)^52^ was used to assess gait stability as it is a valid measure of the mechanical stability of the body configuration during large balance perturbations^53,54^. It was hypothesised that healthy young adults would demonstrate reactive adaptation of gait following repeated gait perturbations, that these adaptations would be partly retained 1 month later, that evidence of savings in both the acute response to a single perturbation and in the recovery behaviour over multiple perturbations would be found, and that the adaptation to repeated perturbations to one lower limb would transfer and benefit gait stability following perturbations to the contralateral lower limb, as the recovery requires a bipedal response that may be generalisable.

The results of the current study show that young healthy adults can adapt their gait in a reactive, feedback-driven manner and reduce the number of steps required to recover balance following unexpected perturbations to gait and retain these adaptations over a 1-month period. Combined retention and savings led to further improvements in reactive stability control during the second measurement 1 month later. Evidence of interlimb transfer of reactive gait adaptations was inconclusive. Our findings suggest that young healthy adults utilise retention and savings in reactive gait adaptations to benefit stability, but that improvements in stability following perturbations to the untrained limb may not be exclusively due to interlimb transfer of adaptations.

## Results

### Study overview

**Fig. 1:**
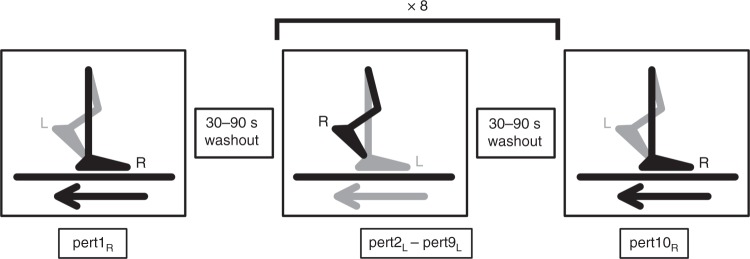
Gait perturbation protocol. The right leg (R) was perturbed by the treadmill belt acceleration first (pert1_R_), followed by eight perturbations (pert2_L_ – pert9_L_) to the left leg (L), and the final perturbation (pert10_R_) was again applied to the right leg (R). In all, 30–90 s time of unperturbed walking occurred between each perturbation. The perturbation consisted of a 3 m s^−^^2^ acceleration of the treadmill belt to 180% of the stability-normalised walking speed, triggered automatically when the vertical projection of the hallux marker of the to-be-perturbed limb became anterior to the hallux marker of the stance limb in the sagittal plane. The perturbation was designed to cause a forward rotation and acceleration of the upper body, relative to the lower body, leading to a forward loss of dynamic stability

**Fig. 2:**
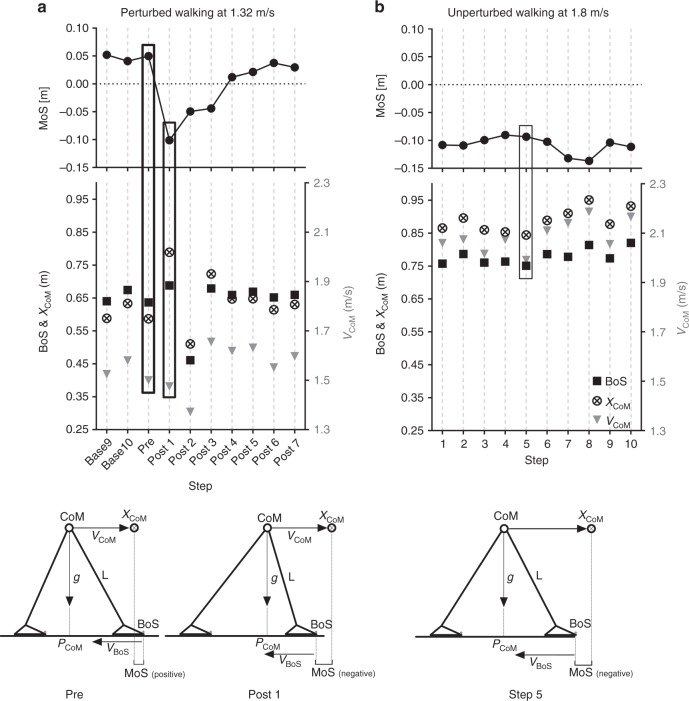
The margin of stability (MoS) components during perturbed and unperturbed walking. Representative data from one individual participant during a perturbation (**a**) and during fast unperturbed walking (**b**), as well as schematic representations of the body configurations and MoS components at foot touchdown of the step before the perturbation (Pre), the first step post-perturbation (Post1) and for one step during fast unperturbed walking that elicited a similar MoS to Post1 (Step 5). The anteroposterior MoS were calculated for the moment of foot touchdown as the anteroposterior difference between the base of support (BoS; anteroposterior distance between the hallux markers) and the extrapolated centre of mass (*X*_CoM_) as defined by Hof et al.^52^, adapted for the reduced kinematic model^55^. The BoS, *X*_CoM_ and MoS are indicated on the left *y* axes, and the velocity of the centre of mass (*V*_CoM_) on the right *y* axes. The BoS and *X*_CoM_ positions are relative to the posterior hallux marker position in the anteroposterior direction. Note that while Step 5 displays a comparable MoS value to Post1, the absolute and relative positions of the components of the MoS are not all the same, indicating that the change in belt velocity during the perturbation is not the sole reason for the change in MoS

In order to test our hypotheses, 18 healthy young adult participants were subjected to 10 unilateral treadmill belt accelerations during walking on 1 day, as shown in Fig. 1 (see Methods for details). The participants returned approximately 1 month later (28.4 ± 3.4 days) and repeated the perturbation protocol, although the participants were only aware that they would complete a “walking balance challenge” and were told that it could be different on the second day. The gait perturbation protocol was conducted at a stability-normalised walking speed based on trials of unperturbed walking at various speeds for each individual, to ensure that all participants were walking at comparable stability levels^50^. The stability-normalised walking speeds ranged from 1.22 m s^−1^ to 1.51 m s^−1^ with a mean ± SD of 1.33 ± 0.07 m s^−1^. In order to quantify stability, we determined the anteroposterior MoS at the moment of foot touchdown as defined by Hof et al.^52^, adapted for a reduced kinematic model based on Süptitz et al.^55^. Representative data from one participant during a perturbation and during fast walking is shown in Fig. 2, alongside schematic representations of the body configuration at specific time points, to illustrate how the components of the MoS are affected by different walking conditions.

In the following results, data are presented as median and 95% confidence intervals unless otherwise stated. Day 1 values are represented by filled symbols, Day 2 values by empty symbols. Perturbations to the right leg are represented by squares and perturbations to the left leg by circles. Perturbations of the same number (i.e., Pert1_R_) are represented by the same colours. The data used to create each figure can be found in Supplementary Data 1.

### Reactive gait adaptations to repeated perturbations

**Fig. 3:**
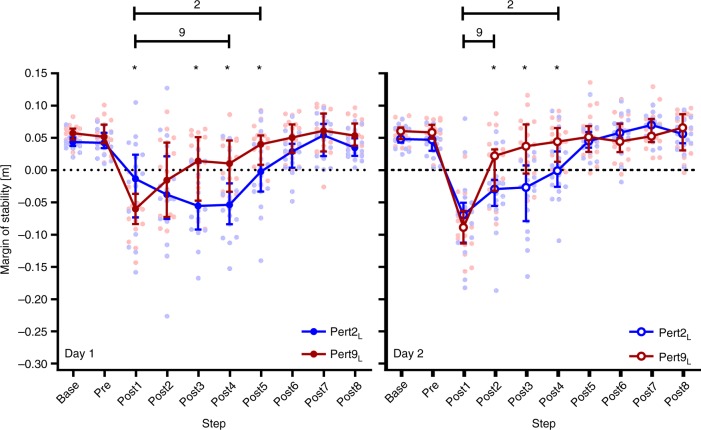
Reactive adaptations in gait during repeated perturbations. Median and 95% confidence intervals of the margins of stability during the second and ninth perturbations (the first and final perturbation of the left leg; Pert2_L_, and Pert9_L_, respectively) during unperturbed walking prior to each perturbation (Base), the final step prior to each perturbation (Pre) and the first eight recovery steps following the perturbations (Post1-8) on Day 1 (left panel) and Day 2 (right panel) of the measurements. *Significant difference between Pert2_L_ and Pert9_L_ (*P* < 0.05). Lines with 2 and 9: all steps under the line were significantly different to Base for Pert2_L_, and Pert9_L_, respectively (*P* < 0.05)

The two-way repeated-measures analysis of variances (ANOVAs) revealed significant perturbation number and step effects and significant perturbation number by step interactions on the MoS for Day 1 (*F*_[3,51]_ = 7.117, *P* = 0.0004; *F*_[9,153]_ = 39.05, *P* < 0.0001; and *F*_[27, 459]_ = 2.788, *P* < 0.0001, respectively) and Day 2 (*F*_[3,51]_ = 14.69, *P* < 0.0001; *F*_[9,153]_ = 49.11, *P* < 0.0001; and *F*_[27,459]_ = 5.943, *P* < 0.0001, respectively). Tukey’s multiple comparisons tests revealed that MoS during Base and Pre were not significantly affected by perturbation number (0.30 < *P* < 0.99; see Supplementary Table 1). Regarding the adaptation of gait on Day 1, the participants were able to return to MoS Base values after five and four post-perturbation steps for Pert2_L_ and Pert9_L_, respectively, indicated by MoS values significantly different to Base for Post1-5 and Post1-4, respectively (Fig. 3; for detailed multiple comparisons results, see Supplementary Table 2). On Day 2, further adaptation across the left leg perturbations was seen as Pert9_L_ required only two recovery steps for participants to regain stability, compared with four steps during Pert2_L_, indicated by MoS values significantly different to Base for Post1-2 and Post1-4, respectively (Fig. 3; Supplementary Table 3).

### Retention of reactive adaptations in gait

**Fig. 4:**
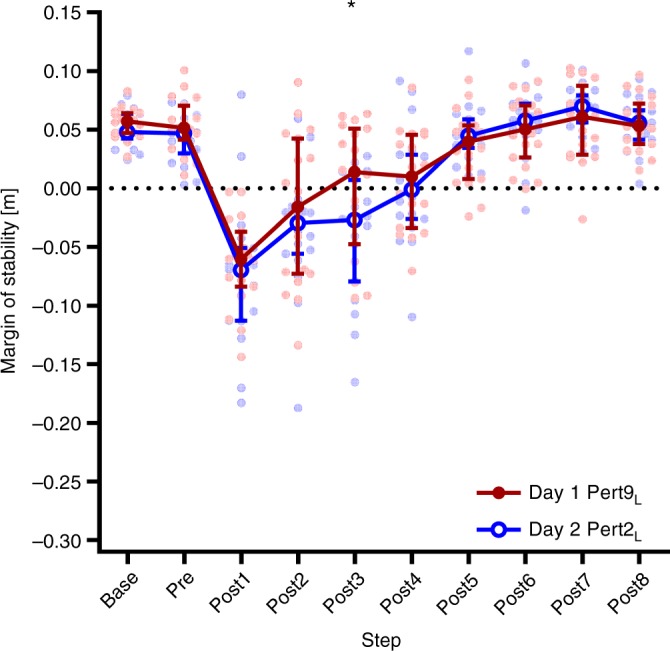
Retention of reactive adaptations in gait. Median and 95% confidence intervals of the margins of stability during the ninth perturbation on Day 1 and the second perturbation on Day 2, representing the final and first perturbations to the left leg on Day 1 and Day 2, respectively (Day 1 Pert9_L_ and Day 2 Pert2_L_, respectively) during unperturbed walking prior to each perturbation (Base), the final step prior to each perturbation (Pre) and the first eight recovery steps following the perturbations (Post1-8). *Significant difference at the indicated step between Day 1 Pert9_L_ and Day 2 Pert2_L_ (*P* = 0.0002)

Regarding retention of the Day 1 adaptations to perturbations of the left leg after 1 month, Pert2_L_ on Day 2 resulted in participants requiring the same number of recovery steps (four) before returning to MoS Base as during Pert9_L_ on Day 1 (Fig. 4 and Supplementary Tables 2 and 3). A direct comparison of Day 1 Pert9_L_ vs. Day 2 Pert2_L_ revealed a significant perturbation number by step interaction (*F*_[9,153]_ = 2.696, *P* = 0.0061) and the post-hoc comparisons revealed significant differences for Post3 only (*P* = 0.0002; Fig. 4).

### Interlimb transfer and savings of gait adaptations

**Fig. 5:**
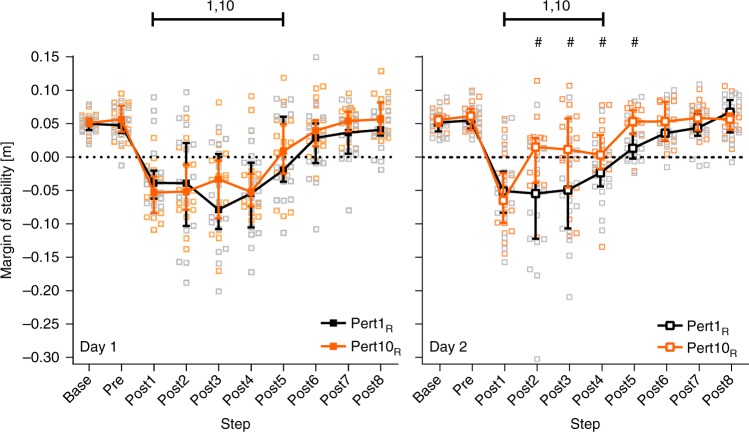
Interlimb transfer of reactive adaptations in gait. Median and 95% confidence intervals of the margins of stability during the first and tenth perturbations (the first and final perturbation of the right leg; Pert1_R_, and Pert10_R_, respectively) during unperturbed walking prior to each perturbation (Base), the final step prior to each perturbation (Pre) and the first eight recovery steps following the perturbations (Post1-8) on Day 1 (left panel) and Day 2 (right panel) of the measurements. ^#^Significant difference between Pert1_R_ and Pert10_R_ (*P* < 0.05). Lines with 1 and 10: all steps under the line were significantly different to Base for Pert1_R_ and Pert10_R_, respectively (*P* < 0.05)

The adaptation to perturbations applied to the left leg did not appear to transfer to stability recovery following perturbations to the right leg on Day 1, as no significant differences were found between Pert1_R_ and Pert10_R_ for any step (Fig. 5; also see Supplementary Table 4) and the number of steps needed post-perturbation to return to MoS Base was the same during Pert1_R_ and Pert10_R_ (Fig. 5; also see Supplementary Table 2). However, Pert1_R_ on Day 2 required one step less in order to recover to MoS Base, compared with Day 1 Pert1_R_ and Pert10_R_ (Fig. 5 and Supplementary Table 3). In contrast, the adaptation to perturbations applied to the left leg on Day 2 did appear to transfer and benefit stability recovery following perturbations to the right leg, as significant differences were found between Pert1_R_ and Pert10_R_ for Post2-5 (Fig. 5; also see Supplementary Table 5), although the number of steps needed post-perturbation to return to MoS Base was the same (four) during Pert1_R_ and Pert10_R_ (Fig. 5; also see Supplementary Table 3). To further investigate the results regarding interlimb transfer of reactive adaptations in gait, post-hoc analyses were conducted (see below).

The presence of savings was unclear, due to the almost complete retention of adaptations on Day 2 (Pert2_L_ Day 2 vs. Pert9_L_ Day 1; Figs. 3 and 4; Supplementary Tables 2 and 3). Post-hoc analyses were conducted to further investigate savings (see below).

### Post-hoc analyses of savings and interlimb transfer

**Fig. 6:**
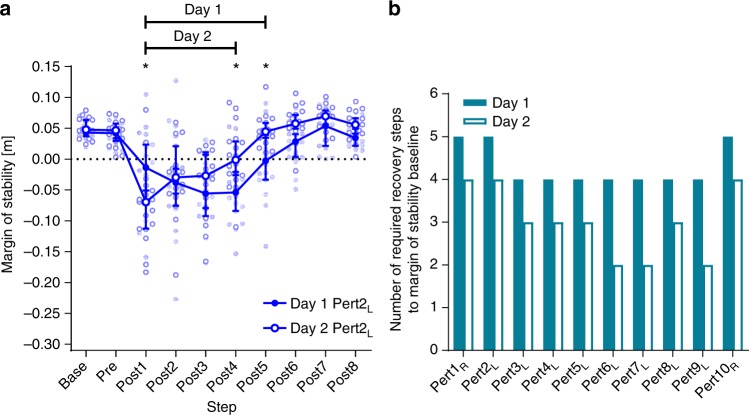
Savings in reactive adaptations in gait. **a** Median and 95% confidence intervals of the margins of stability during the first perturbation to the left leg on Day 1 and Day 2 (Day 1 Pert2_L_ and Day 2 Pert2_L_, respectively) during unperturbed walking prior to each perturbation (Base), the final step prior to each perturbation (Pre) and the first eight recovery steps following the perturbations (Post1-8). *Significant difference between Day 1 and Day 2 (*P* < 0.05). Lines with Day 1 and Day 2: all steps under the line were significantly different to Base for Day 1 and Day 2, respectively (*P* < 0.05). **b** The number of steps required during each of the ten perturbations on Day 1 and Day 2 to return to baseline margins of stability as assessed by two-way repeated-measures ANOVAs with Bonferroni’s multiple comparisons tests

As full retention was seen in the number of steps to recover to MoS Base in Day 2 Pert2_L_, compared with Day 1 Pert9_L_, it was unclear from the pre-planned analysis if savings in the recovery response were present. To investigate the possible presence of savings in the acute recovery response, Pert2_L_ from each day was analysed in a two-way repeated-measures ANOVA with day and step as factors, with Bonferroni’s test for multiple comparisons. This analysis revealed evidence of savings, as the rate of recovery to MoS Base was significantly faster (savings), with significant post-hoc differences between Pert2_L_ on Days 1 and 2 at Post4 and Post5 (Fig. 6a and Supplementary Table 6). To further investigate savings in the overall recovery response, two-way repeated-measures ANOVAs with step and perturbation number as factors with Bonferroni’s test for multiple comparisons were conducted for all perturbations on Day 1 and Day 2, and revealed that the number of steps required to reach MoS baseline after the perturbations plateaued at four steps from the third perturbation onwards on Day 1, while on Day 2, as little as two steps where required by the sixth perturbation (Fig. 6b and Supplementary Table 7). The numbers of steps to return to MoS baseline are summarised in Fig. 6b, and the full results of these ANOVAs can be found in Supplementary Table 7.

**Fig. 7:**
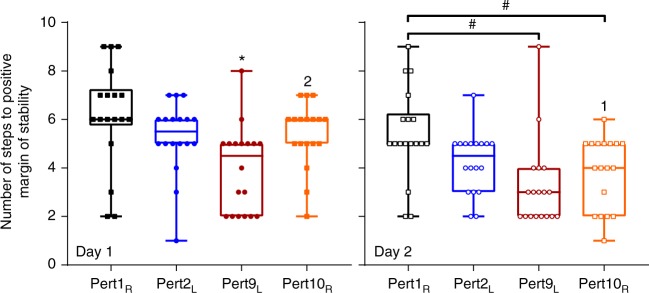
Number of steps to positive margins of stability. Box plots and individual data points of the number of steps required to reach positive margins of stability following the first and final perturbation to each lower limb (Pert1_R_, Pert2_L_, Pert9_L_ and Pert10_R_) on each day for each participant. *Significantly different to all other perturbations on that day (*P* < 0.05). ^#^Significant difference between the indicated perturbations (*P* < 0.01). 1 or 2: Significant difference to that same perturbation number on Day 1 or Day 2, respectively (*P* < 0.01)

The pre-planned analysis appeared to reveal evidence of interlimb transfer on Day 2, but not Day 1. In order to explore these findings further, we calculated the number of steps required to reach consistently positive MoS values following Pert1_R_, Pert2_L_, Pert9_L_ and Pert10_R_ on each day, for each individual. A two-way repeated-measures ANOVA with day and perturbation number as factors with Bonferroni’s test for multiple comparisons revealed significant day (*F*_[1,17]_ = 8.951, *P* = 0.0082) and perturbation number (*F*_[3,51]_ = 15.79, *P* < 0.0001) effects on the number of steps to reach positive MoS (Fig. 7). Regarding interlimb transfer, Pert10_R_ on Day 2 required significantly fewer steps for participants to reach positive MoS values compared with Pert1_R_ on Day 2 (*P* = 0.0016) and Pert10_R_ on Day 1 (*P* = 0.0016; Fig. 7).

**Fig. 8:**
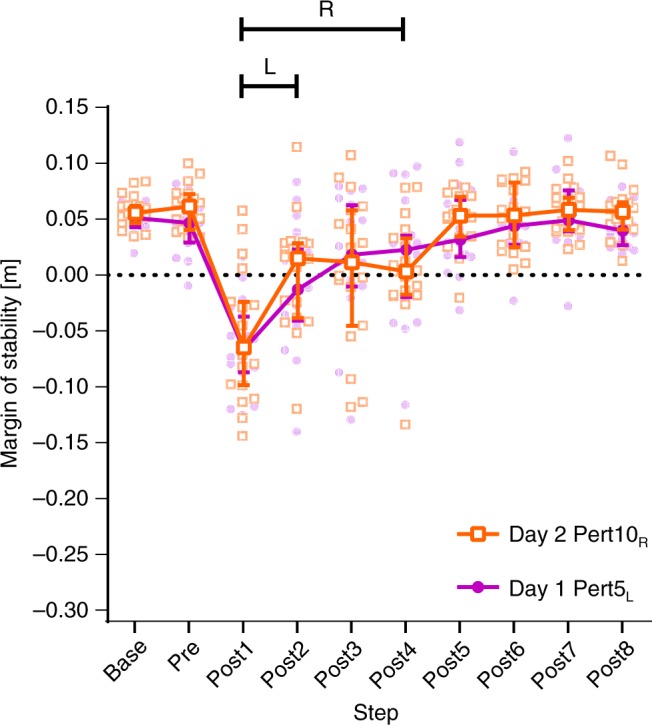
Comparison of the reactive responses to the fourth perturbation of each limb. Median and 95% confidence intervals of the margins of stability during the fourth perturbation to each leg (Day 1 Pert5_L_ and Day 2 Pert10_R_, respectively) during unperturbed walking prior to each perturbation (Base), the final step prior to each perturbation (Pre) and the first eight recovery steps following the perturbations (Post1-8). Lines with R and L: all steps under the line were significantly different to Base for the right and left limb, respectively (*P* < 0.05)

To determine if the apparent interlimb transfer of adaptations in stability on Day 2 (see Figs. 5 and 7) were purely due to transfer, or partly due to a practice effect of the right leg, a two-way repeated-measures ANOVA with step and perturbation number as factors with Bonferroni’s test for multiple comparisons were conducted for the fourth perturbation to each leg: Day 1 Pert5_L_ and Day 2 Pert10_R_, respectively. No significant effect of perturbation number was found. However, during Day 1 Pert5_L_, three steps were needed to return to baseline MoS, whereas during Day 2 Pert10_R_, five steps were needed (Fig. 8).

## Discussion

In this study, we assessed the reactive adaptation of gait in response to repeated gait perturbations in young healthy adults, how this adaptation was retained after 1 month (28.4 ± 3.4 days), and if savings and interlimb transfer of these adaptations could be observed. We hypothesised that healthy young adults would demonstrate reactive adaptation of gait following repeated gait perturbations, that these adaptations would be partly retained 1 month later, that evidence of savings in both the acute response to a single perturbation and in the general recovery behaviour over multiple perturbations would be found, and that evidence of interlimb transfer of adaptations in stability would be found. The first and second hypotheses regarding adaptation and retention were confirmed, as significant improvements in MoS and number of steps to MoS Base during the perturbations to the left leg on the first day were observed, and these improvements were (almost completely) retained during the first perturbation to the left leg on the second measurement day (post 1 month), confirming previous work demonstrating adaptation and retention in reactive gait stability^4,6,8–10^. The third hypothesis regarding savings was confirmed in post-hoc analyses, as the pre-planned analysis could not confirm this due to the unexpected extent of the retention observed. Finally, our hypothesis that evidence of interlimb transfer of adaptations in gait stability would be observed was not conclusively supported nor refuted. No clear difference between stability during the two right leg perturbations on Day 1 were seen, but improvements in the recovery during the right leg perturbations on Day 2 were found. However, we could not conclusively determine if the gait adaptations observed during the right leg perturbations on Day 2 were strictly due to interlimb transfer or to independent or combined effects of interlimb transfer, perturbation repetition, and task awareness.

The current study deals with reactive (feedback-driven) adaptations in gait, and how these are retained, saved and transferred. We must first confirm that these adaptations were indeed predominantly reactive, and that they were not significantly influenced by predictive (feedforward) adaptations in gait. Predictive adaptations in gait did not occur, at least not in a way that influenced the MoS, as Base and Pre were not significantly affected by perturbation number (Supplementary Table 1). This may be, in part due to our perturbation paradigm, as no visual cues occurred for these treadmill delivered perturbations, as may be the case in overground situations^5^ and the timing between perturbations was variable and unpredictable. Taking these considerations together, we assume that the results represent predominately reactive adaptations in gait, and were not due to predictive adjustments. Previous studies have also found independent adaptations in reactive stability control^9,56–59^. However, the current study design does not allow us to determine how much of the adaptations observed in gait stability control can be attributed to direct modulation of spinal reflexes as seen in response to simulated trip perturbations in infants prior to independent walking^38,40^. In the current setup, a number of these reflexes may have been adapted, as treadmill belt accelerations have been shown to induce stretch reflexes of the plantar flexor muscles^60^ and interlimb reflexes^61^. Such reflexes can also be modulated based on the walking environment and the potential threat to gait stability^32,62^, meaning that once the participants in the current study had experienced a perturbation, certain reflexes may have been modulated to elicit faster or greater responses, which could partly explain the improved gait stability and further adaptation observed on Day 2. Given this evidence, it seems reasonable to assume that the adaptation of gait observed in the current study could be, at least in part, due to feedback-driven adaptations of spinal reflexes that are important for gait stability.

**Fig. 9:**
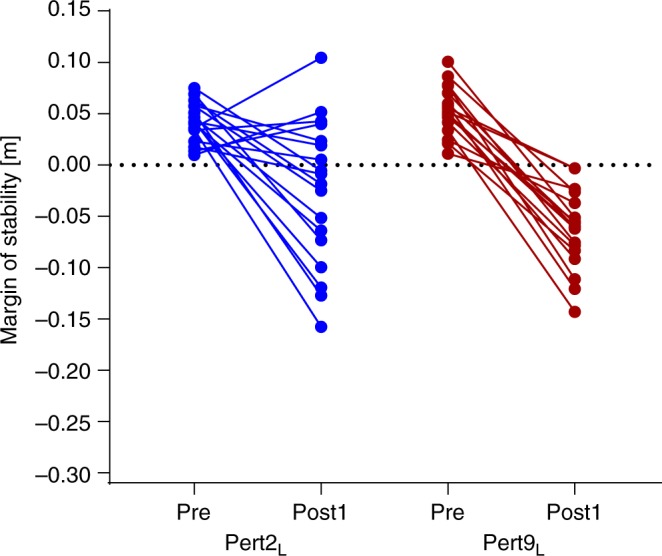
Individual responses to Day 1 perturbations. Individual margins of stability values for the steps immediately before (Pre) and after (Post1) the first and final perturbation to the left leg on Day 1 (Pert2_L_ and Pert9_L_, respectively). Four individuals demonstrated an increase in margins of stability at Post1 during Pert2_L_, but not after repetition of the perturbations

An interesting outcome on Day 1 was that some participants demonstrated an increase, rather than a decrease in stability at Post1 during Pert2_L_ (see Fig. 9 for the individual values). With repetition of the perturbations, participants adapted towards a decrease in stability (Pert9_L_, Fig. 9), which was maintained on Day 2. At first glance, this change does not appear logical; why would participants decrease their stability with practice? On closer inspection of the data and of the video recordings, it appears that some individuals created a large increase in the base of support with a large anterior step, to prevent a forward loss of balance, resulting in an increase in the MoS. However, as the treadmill was moving at a fixed speed, participants then had to “catch up” with the treadmill belt. This strategy appears to initially prioritise stability control and neglect the secondary task of continuing walking.

Our results showed that young healthy adults are capable of almost fully retaining reactive adaptations in gait over a period of 1 month. Previous work has repeatedly demonstrated partial retention of reactive gait adaptations in healthy adults^8–10,49^. The reasons why our participants demonstrate almost full retention are unclear, but could be related to the nature of the perturbation, the normalisation procedure or awareness of the task. What we can conclude from these results is that young adults do not necessarily need frequent or consecutive exposure to unexpected gait perturbations to improve their reactive gait stability. This finding aligns with one recent study that showed that older adults need only one overground slip perturbation to trigger beneficial, long-lasting adaptations in stability control^49^.

In contrast to our hypothesis, no interlimb transfer of adaptations in reactive gait stability appeared to occur on Day 1. This result was surprising for two reasons. First, although the perturbations were applied to one leg specifically, multiple recovery steps are needed following such perturbations to control balance (i.e., both legs are necessary for recovery), as well as upper body control and counter rotations^63^. Therefore, we suspected that the response during and immediately following each perturbation (i.e., the first recovery step) may be limb specific, due to different requirements for braking and propulsion similar to previous suggestions for slip recovery^64^, but that the following alterations in gait would be generalisable and could consequently benefit stability control. Second, as opposed to most previous studies on interlimb transfer in gait (of which one has shown that adaptations transfer across limbs^65^ while others have not^2,66,67^), our paradigm required reactive adaptation to repeated, unexpected perturbations, not feedforward correction of errors during continuous perturbation. Abrupt, as opposed to gradual, gait perturbations provide a substantial amount of error feedback that can aid participants’ gait adaptation^3,68,69^ and savings^70^. Based on this evidence, we reasoned that the adaptations in stability control seen after the perturbations of the left leg may be transferred to aid recovery from perturbation to the right leg. One previous study that did analyse interlimb transfer of adaptations in reactive gait stability found that participants could only transfer pre-perturbation adaptations (predictive, not reactive) in gait between limbs^64^, but their analysis only included the first recovery step and the perturbation was not standardised, meaning that the exact impact of the perturbation to each limb may have slightly differed. Regarding the improvements in stability observed following the two perturbations to the right leg on Day 2, we cannot conclusively say, based on the current data, whether or not these were due to interlimb transfer of adaptations. Our post-hoc analysis seems to suggest that a practice effect may have contributed to these findings (Fig. 8), but not all of the first four perturbations to each limb occurred on the same day, limiting the conclusions that can be drawn.

As can be seen in our results, individual responses to the perturbations varied. It could be argued that individual variability within and between participants may therefore influence the analysis and that by averaging repeated trials, a clearer picture of the effect of the perturbations could be gained. However, as the effects of these perturbations are so strong, we do not feel that this variability compromises the study. Analysing these initial single trials could be considered a strength in terms of ecological validity, as the variation in responses is more representative of what is seen in daily life following real, truly unexpected perturbations to gait^71^.

The participants in the current study were not given any details about the nature of the perturbations, but we did consider the possibility that performance on Day 2 could be influenced by prior knowledge and experience of the task acquired on Day 1. Even though no measurable changes in gait stability during baseline walking were found in the current study, previous studies have demonstrated the beneficial effects of increased awareness of perturbations on stability recovery performance following trips^72,73^. For the eight perturbations to the left leg, the plateau of recovery steps required for re-stabilisation on Day 1 was quickly improved upon on Day 2 (Figs. 6b and 7). It is unclear if this was due to independent or combined effects of retention, savings or increased task awareness, but we can conclude that for this form of gait perturbation, “complete” adaptation on first exposure does not necessarily represent the participant’s best task performance, which has implications for perturbation-based balance training programmes.

In conclusion, we have shown that young healthy adults are capable of adapting their gait in a reactive, feedback-driven manner to control stability and reduce the number of steps to reach positive and baseline values of MoS, and that they can fully retain these adaptations over a 1-month period. On re-exposure to the perturbations, a combination of retention and savings led to further improvements in reactive stability control above those made 1 month before. In contrast to our expectations, evidence of interlimb transfer of reactive gait adaptations was inconclusive. Our results show that humans utilise retention and savings in reactive gait adaptations to benefit stability, but that interlimb transfer may not be exclusively responsible for improvements following perturbations to the untrained limb. These findings broaden our understanding of reactive gait adaptability and have implications for future studies on gait stability and adaptability, as well as for falls prevention interventions.

## Methods

### Participants

Eighteen healthy adults participated in this study (eight males, 10 females; age: 24.4 ± 2.5 years; height: 174.9 ± 7.4 cm; weight: 74.6 ± 15.2 kg). The participants had no self-reported history of walking difficulties, dizziness or balance problems, and had no known neuromuscular condition or injury that could affect balance or walking. Informed consent was obtained and the study was conducted in accordance with the Declaration of Helsinki. The study protocol was approved by the Maastricht University Medical Centre medical ethics committee (NL58205.068.16).

### Setup and procedures

The Computer Assisted Rehabilitation Environment Extended (CAREN; Motekforce Link, Amsterdam, The Netherlands) was used for this study, which included a dual-belt force plate-instrumented treadmill (Motekforce Link, Amsterdam, The Netherlands; 1000 Hz), a 12-camera motion capture system (100 Hz; Vicon Motion Systems, Oxford, UK) and a virtual environment that provided optic flow during walking. Three high definition video cameras also recorded video footage of the measurements. A safety harness system connected to an overhead frame was used at all times. Five retroreflective markers were attached to anatomical landmarks (C7, left and right trochanter and left and right hallux) and the three-dimensional coordinates of these markers were tracked by the motion capture system. Each session began with walking familiarisation trials at 0.4 m s^−1^ up to 1.8 m s^−1^. Sixty seconds were used for each speed. Participants were then given sufficient rest (approximately 2 min) before continuing with the measurements.

The procedures for determining the stability-normalised walking speed, as well as the theoretical background and data regarding the effectiveness of this approach are described in detail elsewhere^50^. Briefly, single two-to-three-minute-long measurements were conducted at 0.4 m s^−1^ up to 1.8 m s^−1^ in 0.2 m s^−1^ intervals. During a second rest period for the participants, the stability-normalised walking speed was calculated. In order to determine the stability-normalised walking speed, the mean anteroposterior MoS (see below) at foot touchdown of the final 10 steps of each walking trial (0.4 m s^−1^ to 1.8 m s^−1^) were taken and were fitted with a second-order polynomial function. For each participant, the walking speed that would result in MoS of 0.05 m was calculated from the function.

The gait perturbation protocol then began with 3–4 min of unperturbed walking at the stability-normalised walking speed, in order to allow participants to familiarise themselves with this speed. The participants then experienced 10 unilateral treadmill belt acceleration perturbations, each occurring every 30–90 s without warning (the washout time periods between perturbations was the same for all subjects; Fig. 1). The first and last perturbed the right leg, while the second to ninth repeatedly perturbed the left leg (Fig. 1). The perturbation consisted of a 3 m s^−^^2^ acceleration of the treadmill belt to 180% of the stability-normalised walking speed, triggered automatically (using the D-Flow software of the CAREN; Motekforce Link, Amsterdam, The Netherlands) when the vertical projection of the hallux marker of the to-be-perturbed limb became anterior to the hallux marker of the stance limb in the sagittal plane. Thereby, the belt acceleration started before foot touchdown to allow a higher magnitude of perturbation to the entire stance phase. The belt decelerated when the perturbed limb lost contact with the ground (toe-off; see below). Any consecutive foot contacts with the perturbed belt (i.e., when both the perturbed limb and the first recovery step were accelerated) were noted, but none occurred during the perturbations analysed in this study. The participants returned approximately 1 month later (28.4 ± 3.4 days) and repeated the perturbation protocol. On each occasion, participants were told that they would complete a walking balance challenge lasting about 10–15 min, and that their task was to try to continue walking as normally as possible. The participants were unaware of the specifics of the perturbation protocol (i.e., limbs to be perturbed, type, number, timing, magnitude of the perturbations) and no warnings or cues were given prior to the perturbations. Note that we also made participants aware of the capacities of the CAREN system, in that it could provide perturbations via platform shifts and pitches as well as treadmill belt movements. On the second day, they were informed that they would again experience a walking balance challenge lasting about 10–15 min, but that it could be different to the first day. We asked the participants about what they perceived regarding the perturbations following the first session and no participant was able to describe the precise protocol, suggesting that knowledge of the order of the perturbations would not have influenced the performance on the second measurement day. This study did not account for lower limb dominance, but due to the bipedal nature of the task (i.e., multiple recovery steps are required) and that we observed no significant differences between the first two perturbations (to the left and right lower limbs, respectively), we do not feel that this will have had a meaningful influence on the results. In fact, no previous study has highlighted limb dominance to be a major factor in gait stability, with one study specifically investigating the issue using a forward lean-and-release task and finding no differences between limbs in young and older adults^74^.

### Data processing and MoS calculation

The three-dimensional coordinates of the markers were filtered using a low pass second-order Butterworth filter (zero-phase) with a 12 Hz cut-off frequency. For all steps, the foot marker anteroposterior velocity data were used to determine foot touchdown and toe-off (the frame in which the marker velocity direction switched)^75^. This was then corrected based on the average discrepancy between a force plate-determined touchdown and toe-off (with a force threshold of 50 N) and the marker-determined touchdown and toe-off for all steps that contacted only one force plate. This combined method was used to be able to accurately account for foot touchdowns and toe-offs occurring in the centre of the treadmill triggering both force plates simultaneously. The anteroposterior MoS were calculated for the moment of foot touchdown as the anteroposterior difference between the base of support (anteroposterior distance between the hallux markers) and the extrapolated centre of mass (*X*_CoM_) as defined by Hof et al.^52^, adapted for the reduced kinematic model based on Süptitz et al.^55^:

$${{X}}_{{\mathrm{CoM}}} = \frac{{{{P}}_{{\mathrm{TroL}}} + {{P}}_{{\mathrm{TroR}}}}}{2} - {{P}}_{{\mathrm{HalluxP}}} + \frac{{0.5\left( {\frac{{{{V}}_{{\mathrm{TroL}}} + {{V}}_{{\mathrm{TroR}}}}}{2} + {{V}}_{{\mathrm{C}}7}} \right) + \left| {{{V}}_{{\mathrm{Belt}}}} \right|}}{{\sqrt {\frac{{{g}}}{{{{L}}_{{\mathrm{Ref}}}}}} }}$$

where *P*_Trol_, *P*_Trol_ and *P*_HalluxP_ represent the trochanter and the rearmost hallux marker anteroposterior positions respectively; *V*_Trol_, *V*_TroR_ and *V*_C7_ are the anteroposterior velocities of the trochanter and C7 markers respectively; *V*_Belt_ is the treadmill belt velocity; *g* is gravitational acceleration (9.81 m s^−2^); and *L*_Ref_ is the reference leg length. The MoS concept is one of the few well-defined and well-accepted biomechanical measures of mechanical stability of the body configuration during dynamic movement^54^, with one study demonstrating that, during a forward loss of balance, participants who required multiple recovery steps had a negative MoS value at touchdown of the first recovery step in all cases, whereas participants who only required this one recovery step all had a positive MoS^53^. The MoS was calculated for the following steps: baseline for each perturbation was the mean MoS of the eleventh to second last step before each perturbation (Base); the final step before each perturbation (Pre); and the first eight recovery steps following each perturbation (Post1-8).

### Statistics

Two-way repeated-measures ANOVAs with perturbation number (Pert1_R_, Pert2_L_, Pert9_L_ and Pert10_R_, representing the first and final perturbations to each limb on each day) and step (Base, Pre, Post1-Post8) as factors with post-hoc Tukey’s tests for multiple comparisons were used for each day to determine the following: predictive adaptation across the perturbation protocol (Perturbation number difference in Base and Pre); acute adaptation to the perturbation on each day (Pert2_L_ vs. Pert9_L_); acute interlimb transfer of adaptations on each day (Pert1_R_ vs. Pert10_R_); savings in the acute recovery response to a perturbation (quicker return to baseline MoS in Day 2 Pert2_L_ than Day 1 Pert2_L_). Retention of adaptations over 1 month was investigated with a separate two-way repeated-measures ANOVA with Bonferroni’s multiple comparisons test (Day 1 Pert9_L_ vs. Day 2 Pert2_L_). Normality of the data was checked using the Shapiro–Wilk test and Q-Q plots. In addition to these pre-planned analyses, post-hoc explorative statistical tests were conducted (see Results). Significance was set at *α* = 0.05. Analyses were performed using GraphPad Prism version 7.03 for Windows (GraphPad Software Inc., La Jolla, California, USA).

### Code availability

The code used to process the motion capture data in the current study are available from the corresponding author on reasonable request.

## Supplementary information

Supplementary Information

Supplementary Data 1

Description of Additional Supplementary Files

## Data Availability

The datasets generated and analysed during the current study are available from the corresponding author on reasonable request. Data used for generating the plots in the main figures are available in Supplementary Data 1.
