# Supplementary material for: Retention, savings and interlimb transfer of reactive gait adaptations in humans following unexpected perturbations
Source: Commun Biol. 2018 Dec 14;1:230. doi: 10.1038/s42003-018-0238-9 (PMC6294781; doi:10.1038/s42003-018-0238-9)
Supplement: Supplementary file 1 — Supplementary Information [file 42003_2018_238_MOESM1_ESM.pdf]

Supplementary Table 1. Tukey's multiple comparison test results for Base and Pre margins of stability.

Tukey's multiple comparisons test	Mean Diff. (cm)	95.00% CI of diff. (cm)	Significant?	Adjusted P Value
**Day 1 Base**				
**Pert1$_R$ vs. Pert2$_L$**	0.0048	-0.02653 to 0.03621	No	0.9787
**Pert1$_R$ vs. Pert9$_L$**	-0.0076	-0.03892 to 0.02381	No	0.9254
**Pert1$_R$ vs. Pert10$_R$**	-0.0030	-0.03439 to 0.02834	No	0.9946
**Pert2$_L$ vs. Pert9$_L$**	-0.0124	-0.04376 to 0.01897	No	0.7385
**Pert2$_L$ vs. Pert10$_R$**	-0.0079	-0.03923 to 0.0235	No	0.9167
**Pert9$_L$ vs. Pert10$_R$**	0.0045	-0.02684 to 0.03589	No	0.9824
**Day 1 Pre**				
**Pert1$_R$ vs. Pert2$_L$**	0.0044	-0.02701 to 0.03572	No	0.9843
**Pert1$_R$ vs. Pert9$_L$**	-0.0073	-0.03868 to 0.02405	No	0.9316
**Pert1$_R$ vs. Pert10$_R$**	-0.0088	-0.04017 to 0.02257	No	0.8878
**Pert2$_L$ vs. Pert9$_L$**	-0.0117	-0.04304 to 0.01969	No	0.7725
**Pert2$_L$ vs. Pert10$_R$**	-0.0131	-0.04452 to 0.01821	No	0.7011
**Pert9$_L$ vs. Pert10$_R$**	-0.0015	-0.03285 to 0.02988	No	0.9994
**Day 2 Base**				
**Pert1$_R$ vs. Pert2$_L$**	0.0012	-0.02562 to 0.02796	No	0.9995
**Pert1$_R$ vs. Pert9$_L$**	-0.0066	-0.03342 to 0.02016	No	0.9196
**Pert1$_R$ vs. Pert10$_R$**	-0.0057	-0.03247 to 0.02111	No	0.9474
**Pert2$_L$ vs. Pert9$_L$**	-0.0078	-0.03459 to 0.01899	No	0.8764
**Pert2$_L$ vs. Pert10$_R$**	-0.0068	-0.03364 to 0.01994	No	0.9123
**Pert9$_L$ vs. Pert10$_R$**	0.0010	-0.02584 to 0.02774	No	0.9997
**Day 2 Pre**				
**Pert1$_R$ vs. Pert2$_L$**	0.0142	-0.01262 to 0.04096	No	0.5229
**Pert1$_R$ vs. Pert9$_L$**	-0.0024	-0.02916 to 0.02443	No	0.9958
**Pert1$_R$ vs. Pert10$_R$**	-0.0039	-0.03072 to 0.02286	No	0.9815
**Pert2$_L$ vs. Pert9$_L$**	-0.0165	-0.04333 to 0.01026	No	0.3845
**Pert2$_L$ vs. Pert10$_R$**	-0.0181	-0.04489 to 0.008693	No	0.3032
**Pert9$_L$ vs. Pert10$_R$**	-0.0016	-0.02836 to 0.02523	No	0.9988

Supplementary Table 2. Tukey's multiple comparison test results (step comparisons) for margins of stability during perturbation recovery on Day 1.

Tukey's multiple comparisons test	Mean Diff. (cm)	95.00% CI of diff. (cm)	Significant?	Adjusted P Value
**Pert1$_R$**				
**Base vs. Pre**	0.0017	-0.03696 to 0.04039	No	>0.9999
**Base vs. Post1**	0.0746	0.03596 to 0.1133	Yes	<0.0001
**Base vs. Post2**	0.0856	0.04695 to 0.1243	Yes	<0.0001
**Base vs. Post3**	0.1139	0.07519 to 0.1525	Yes	<0.0001
**Base vs. Post4**	0.1032	0.06451 to 0.1419	Yes	<0.0001
**Base vs. Post5**	0.0515	0.01287 to 0.09023	Yes	0.0011
**Base vs. Post6**	0.0275	-0.01116 to 0.06619	No	0.4163
**Base vs. Post7**	0.0171	-0.0216 to 0.05576	No	0.9257
**Base vs. Post8**	0.0080	-0.03071 to 0.04665	No	0.9997
**Pert2$_L$**				
**Base vs. Pre**	0.0012	-0.03745 to 0.03991	No	>0.9999
**Base vs. Post1**	0.0690	0.03032 to 0.1077	Yes	<0.0001
**Base vs. Post2**	0.0763	0.0376 to 0.1149	Yes	<0.0001
**Base vs. Post3**	0.0920	0.05332 to 0.1307	Yes	<0.0001
**Base vs. Post4**	0.0921	0.05345 to 0.1308	Yes	<0.0001
**Base vs. Post5**	0.0465	0.007785 to 0.08514	Yes	0.0059
**Base vs. Post6**	0.0189	-0.01978 to 0.05757	No	0.8696
**Base vs. Post7**	-0.0053	-0.04401 to 0.03334	No	>0.9999
**Base vs. Post8**	0.0056	-0.03308 to 0.04427	No	>0.9999
**Pert9$_L$**				
**Base vs. Pre**	0.0020	-0.03672 to 0.04063	No	>0.9999
**Base vs. Post1**	0.1191	0.08043 to 0.1578	Yes	<0.0001
**Base vs. Post2**	0.0743	0.03563 to 0.113	Yes	<0.0001
**Base vs. Post3**	0.0541	0.01537 to 0.09272	Yes	0.0005
**Base vs. Post4**	0.0482	0.009499 to 0.08685	Yes	0.0034
**Base vs. Post5**	0.0204	-0.01826 to 0.0591	No	0.8071
**Base vs. Post6**	0.0066	-0.03207 to 0.04529	No	>0.9999
**Base vs. Post7**	-0.0008	-0.03951 to 0.03785	No	>0.9999
**Base vs. Post8**	0.0000	-0.03866 to 0.03869	No	>0.9999
**Pert10$_R$**				
**Base vs. Pre**	-0.0041	-0.04273 to 0.03462	No	>0.9999
**Base vs. Post1**	0.1018	0.06307 to 0.1404	Yes	<0.0001
**Base vs. Post2**	0.0964	0.05776 to 0.1351	Yes	<0.0001
**Base vs. Post3**	0.0943	0.05568 to 0.133	Yes	<0.0001
**Base vs. Post4**	0.0883	0.04961 to 0.127	Yes	<0.0001
**Base vs. Post5**	0.0446	0.005905 to 0.08326	Yes	0.0103
**Base vs. Post6**	0.0109	-0.02775 to 0.0496	No	0.9965
**Base vs. Post7**	0.0007	-0.03793 to 0.03942	No	>0.9999
**Base vs. Post8**	-0.0087	-0.04742 to 0.02993	No	0.9994

Supplementary Table 3. Tukey's multiple comparison test results (step comparisons) for margins of stability during perturbation recovery on Day 2.

Tukey's multiple comparisons test	Mean Diff. (cm)	95.00% CI of diff. (cm)	Significant?	Adjusted P Value
**Pert1$_R$**				
**Base vs. Pre**	-0.0046	-0.03759 to 0.02849	No	>0.9999
**Base vs. Post1**	0.0987	0.06567 to 0.1317	Yes	<0.0001
**Base vs. Post2**	0.1183	0.08531 to 0.1514	Yes	<0.0001
**Base vs. Post3**	0.1013	0.06826 to 0.1343	Yes	<0.0001
**Base vs. Post4**	0.0760	0.04298 to 0.1091	Yes	<0.0001
**Base vs. Post5**	0.0290	-0.004059 to 0.06202	No	0.1433
**Base vs. Post6**	0.0093	-0.02379 to 0.04229	No	0.9967
**Base vs. Post7**	0.0078	-0.02525 to 0.04082	No	0.9992
**Base vs. Post8**	-0.0082	-0.0412 to 0.02487	No	0.9988
**Pert2$_L$**				
**Base vs. Pre**	0.0085	-0.02459 to 0.04149	No	0.9984
**Base vs. Post1**	0.1238	0.09074 to 0.1568	Yes	<0.0001
**Base vs. Post2**	0.0843	0.05126 to 0.1173	Yes	<0.0001
**Base vs. Post3**	0.0903	0.05724 to 0.1233	Yes	<0.0001
**Base vs. Post4**	0.0480	0.01494 to 0.08102	Yes	0.0002
**Base vs. Post5**	0.0044	-0.02859 to 0.03748	No	>0.9999
**Base vs. Post6**	-0.0060	-0.03907 to 0.02701	No	0.9999
**Base vs. Post7**	-0.0179	-0.05089 to 0.01518	No	0.7847
**Base vs. Post8**	-0.0042	-0.03725 to 0.02882	No	>0.9999
**Pert9$_L$**				
**Base vs. Pre**	-0.0003	-0.03332 to 0.03275	No	>0.9999
**Base vs. Post1**	0.1439	0.1109 to 0.177	Yes	<0.0001
**Base vs. Post2**	0.0541	0.02107 to 0.08714	Yes	<0.0001
**Base vs. Post3**	0.0250	-0.008096 to 0.05798	No	0.328
**Base vs. Post4**	0.0164	-0.01668 to 0.04939	No	0.8601
**Base vs. Post5**	0.0034	-0.02962 to 0.03646	No	>0.9999
**Base vs. Post6**	0.0092	-0.0238 to 0.04227	No	0.9968
**Base vs. Post7**	-0.0030	-0.03604 to 0.03003	No	>0.9999
**Base vs. Post8**	0.0005	-0.03251 to 0.03357	No	>0.9999
**Pert10$_R$**				
**Base vs. Pre**	-0.0028	-0.03584 to 0.03024	No	>0.9999
**Base vs. Post1**	0.1116	0.07857 to 0.1446	Yes	<0.0001
**Base vs. Post2**	0.0548	0.02181 to 0.08789	Yes	<0.0001
**Base vs. Post3**	0.0520	0.01897 to 0.08505	Yes	<0.0001
**Base vs. Post4**	0.0514	0.01837 to 0.08445	Yes	<0.0001
**Base vs. Post5**	0.0077	-0.02535 to 0.04073	No	0.9992
**Base vs. Post6**	0.0056	-0.02747 to 0.0386	No	>0.9999
**Base vs. Post7**	-0.0003	-0.03335 to 0.03273	No	>0.9999
**Base vs. Post8**	0.0011	-0.03196 to 0.03412	No	>0.9999

Supplementary Table 4. Tukey's multiple comparison test results (comparing perturbations to the right leg) for margins of stability during perturbation recovery on Day 1.

Tukey's multiple comparisons test	Mean Diff. (cm)	95.00% CI of diff. (cm)	Significant?	Adjusted P Value
**$Pert1_R$ vs. $Pert10_R$**				
**Base**	-0.0030	-0.03439 to 0.02834	No	0.9946
**Pre**	-0.0088	-0.04017 to 0.02257	No	0.8878
**Post1**	0.0241	-0.007284 to 0.05545	No	0.1972
**Post2**	0.0078	-0.02359 to 0.03915	No	0.9192
**Post3**	-0.0225	-0.05391 to 0.008823	No	0.25
**Post4**	-0.0179	-0.0493 to 0.01344	No	0.4541
**Post5**	-0.0100	-0.04136 to 0.02137	No	0.8441
**Post6**	-0.0196	-0.05098 to 0.01175	No	0.3725
**Post7**	-0.0194	-0.05073 to 0.012	No	0.3842
**Post8**	-0.0197	-0.05111 to 0.01162	No	0.3667

Supplementary Table 5. Tukey's multiple comparison test results (comparing perturbations to the right leg) for margins of stability during perturbation recovery on Day 2.

Tukey's multiple comparisons test	Mean Diff. (cm)	95.00% CI of diff. (cm)	Significant?	Adjusted P Value
**Pert1$_R$ vs. Pert10$_R$**				
**Base**	-0.0057	-0.03247 to 0.02111	No	0.9474
**Pre**	-0.0039	-0.03072 to 0.02286	No	0.9815
**Post1**	0.0072	-0.01958 to 0.03401	No	0.8992
**Post2**	-0.0692	-0.09597 to -0.04239	Yes	<0.0001
**Post3**	-0.0550	-0.08175 to -0.02817	Yes	<0.0001
**Post4**	-0.0303	-0.05708 to -0.003496	Yes	0.0195
**Post5**	-0.0270	-0.05376 to -0.000176	Yes	0.0478
**Post6**	-0.0094	-0.03616 to 0.01743	No	0.8042
**Post7**	-0.0138	-0.04057 to 0.01302	No	0.5472
**Post8**	0.0036	-0.02323 to 0.03036	No	0.9861

Supplementary Table 6. Bonferroni's multiple comparison test results for margins of stability following the first perturbation to the left lower limb on each day.

**Bonferroni's multiple comparisons test**	**Mean Diff. (cm)**	**95.00% CI of diff. (cm)**	**Significant?**	**Adjusted P Value**
**Day 1 Pert2$_L$ - Day 2 Pert2$_L$**				
**Base**	-0.0068	-0.04848 to 0.03482	No	>0.9999
**Pre**	0.0004	-0.04126 to 0.04204	No	>0.9999
**Post1**	0.0479	0.006305 to 0.0896	Yes	0.0126
**Post2**	0.0012	-0.04045 to 0.04284	No	>0.9999
**Post3**	-0.0085	-0.0502 to 0.0331	No	>0.9999
**Post4**	-0.0510	-0.09263 to -0.009332	Yes	0.0061
**Post5**	-0.0488	-0.0905 to -0.007202	Yes	0.0102
**Post6**	-0.0317	-0.0734 to 0.009895	No	0.3193
**Post7**	-0.0194	-0.06101 to 0.02229	No	>0.9999
**Post8**	-0.0166	-0.05829 to 0.02501	No	>0.9999

Supplementary Table 7. Bonferroni's multiple comparison test results for margins of stability following all 10 perturbations on each day.

	Day 1				Day 2			
**Bonferroni's multiple comparisons test**	**Mean Diff. (cm)**	**95.00% CI of diff. (cm)**	**Significant?**	**Adjusted P Value**	**Mean Diff. (cm)**	**95.00% CI of diff. (cm)**	**Significant?**	**Adjusted P Value**
**Pert1$_R$**								
**Base vs. Pre**	0.0017	-0.03333 to 0.03676	No	>0.9999	-0.0046	-0.03453 to 0.02543	No	>0.9999
**Base vs. Post1**	0.0746	0.0396 to 0.1097	Yes	<0.0001	0.0987	0.06873 to 0.1287	Yes	<0.0001
**Base vs. Post2**	0.0856	0.05059 to 0.1207	Yes	<0.0001	0.1183	0.08837 to 0.1483	Yes	<0.0001
**Base vs. Post3**	0.1139	0.07883 to 0.1489	Yes	<0.0001	0.1013	0.07132 to 0.1313	Yes	<0.0001
**Base vs. Post4**	0.1032	0.06814 to 0.1382	Yes	<0.0001	0.0760	0.04604 to 0.106	Yes	<0.0001
**Base vs. Post5**	0.0515	0.01651 to 0.08659	Yes	<0.0001	0.0290	-0.0009996 to 0.05896	No	0.0729
**Base vs. Post6**	0.0275	-0.007531 to 0.06255	No	0.4685	0.0093	-0.02073 to 0.03923	No	>0.9999
**Base vs. Post7**	0.0171	-0.01796 to 0.05212	No	>0.9999	0.0078	-0.02219 to 0.03776	No	>0.9999
**Base vs. Post8**	0.0080	-0.02707 to 0.04301	No	>0.9999	-0.0082	-0.03814 to 0.02181	No	>0.9999
**Pert2$_L$**								
**Base vs. Pre**	0.0012	-0.03381 to 0.03627	No	>0.9999	0.0085	-0.02153 to 0.03843	No	>0.9999
**Base vs. Post1**	0.0690	0.03395 to 0.104	Yes	<0.0001	0.1238	0.0938 to 0.1538	Yes	<0.0001
**Base vs. Post2**	0.0763	0.04123 to 0.1113	Yes	<0.0001	0.0843	0.05432 to 0.1143	Yes	<0.0001
**Base vs. Post3**	0.0920	0.05695 to 0.127	Yes	<0.0001	0.0903	0.0603 to 0.1203	Yes	<0.0001
**Base vs. Post4**	0.0921	0.05708 to 0.1272	Yes	<0.0001	0.0480	0.018 to 0.07796	Yes	<0.0001
**Base vs. Post5**	0.0465	0.01142 to 0.0815	Yes	0.0007	0.0044	-0.02554 to 0.03442	No	>0.9999
**Base vs. Post6**	0.0189	-0.01615 to 0.05394	No	>0.9999	-0.0060	-0.03601 to 0.02395	No	>0.9999
**Base vs. Post7**	-0.0053	-0.04037 to 0.02971	No	>0.9999	-0.0179	-0.04783 to 0.01212	No	>0.9999
**Base vs. Post8**	0.0056	-0.02945 to 0.04064	No	>0.9999	-0.0042	-0.03419 to 0.02576	No	>0.9999
**Pert3$_L$**								
**Base vs. Pre**	0.0046	-0.03047 to 0.03961	No	>0.9999	0.0034	-0.02656 to 0.0334	No	>0.9999
**Base vs. Post1**	0.0968	0.06175 to 0.1318	Yes	<0.0001	0.1285	0.09848 to 0.1584	Yes	<0.0001
**Base vs. Post2**	0.0521	0.01708 to 0.08716	Yes	<0.0001	0.0689	0.03894 to 0.09889	Yes	<0.0001
**Base vs. Post3**	0.0481	0.01305 to 0.08313	Yes	0.0004	0.0525	0.02256 to 0.08252	Yes	<0.0001
**Base vs. Post4**	0.0594	0.02436 to 0.09444	Yes	<0.0001	0.0290	-0.001018 to 0.05894	No	0.0734

**Base vs. Post5**	0.0222	-0.01288 to 0.05721	No	>0.9999	-0.0017	-0.03172 to 0.02824	No	>0.9999
**Base vs. Post6**	0.0040	-0.03102 to 0.03906	No	>0.9999	0.0007	-0.02924 to 0.03072	No	>0.9999
**Base vs. Post7**	-0.0059	-0.04096 to 0.02913	No	>0.9999	-0.0046	-0.03458 to 0.02537	No	>0.9999
**Base vs. Post8**	0.0034	-0.03164 to 0.03844	No	>0.9999	-0.0024	-0.03241 to 0.02754	No	>0.9999
**$Pert4_L$**								
**Base vs. Pre**	0.0117	-0.02337 to 0.04671	No	>0.9999	0.0064	-0.02353 to 0.03643	No	>0.9999
**Base vs. Post1**	0.0887	0.05371 to 0.1238	Yes	<0.0001	0.1305	0.1005 to 0.1605	Yes	<0.0001
**Base vs. Post2**	0.0532	0.01817 to 0.08826	Yes	<0.0001	0.0667	0.03673 to 0.09668	Yes	<0.0001
**Base vs. Post3**	0.0458	0.01073 to 0.08081	Yes	0.0009	0.0504	0.02042 to 0.08037	Yes	<0.0001
**Base vs. Post4**	0.0477	0.01267 to 0.08275	Yes	0.0004	0.0268	-0.003183 to 0.05677	No	0.1597
**Base vs. Post5**	0.0126	-0.02243 to 0.04766	No	>0.9999	-0.0003	-0.03027 to 0.02969	No	>0.9999
**Base vs. Post6**	0.0132	-0.02188 to 0.0482	No	>0.9999	0.0008	-0.0292 to 0.03076	No	>0.9999
**Base vs. Post7**	-0.0079	-0.0429 to 0.02719	No	>0.9999	-0.0116	-0.04154 to 0.01841	No	>0.9999
**Base vs. Post8**	0.0044	-0.03064 to 0.03944	No	>0.9999	0.0001	-0.02988 to 0.03008	No	>0.9999
**$Pert5_L$**								
**Base vs. Pre**	0.0071	-0.0279 to 0.04218	No	>0.9999	0.0069	-0.02303 to 0.03693	No	>0.9999
**Base vs. Post1**	0.1179	0.08282 to 0.1529	Yes	<0.0001	0.1207	0.09077 to 0.1507	Yes	<0.0001
**Base vs. Post2**	0.0606	0.02553 to 0.09562	Yes	<0.0001	0.0704	0.04042 to 0.1004	Yes	<0.0001
**Base vs. Post3**	0.0399	0.004912 to 0.075	Yes	0.0091	0.0431	0.01313 to 0.07309	Yes	0.0001
**Base vs. Post4**	0.0365	0.00145 to 0.07154	Yes	0.0309	0.0241	-0.005919 to 0.05404	No	0.3972
**Base vs. Post5**	0.0105	-0.0245 to 0.04558	No	>0.9999	-0.0065	-0.03643 to 0.02353	No	>0.9999
**Base vs. Post6**	0.0078	-0.02727 to 0.04281	No	>0.9999	0.0008	-0.02922 to 0.03073	No	>0.9999
**Base vs. Post7**	-0.0046	-0.03969 to 0.03039	No	>0.9999	-0.0007	-0.03071 to 0.02925	No	>0.9999
**Base vs. Post8**	0.0094	-0.02559 to 0.04449	No	>0.9999	0.0093	-0.02072 to 0.03923	No	>0.9999
**$Pert6_L$**								
**Base vs. Pre**	0.0069	-0.02811 to 0.04198	No	>0.9999	0.0067	-0.02326 to 0.03669	No	>0.9999
**Base vs. Post1**	0.0921	0.05703 to 0.1271	Yes	<0.0001	0.1174	0.08741 to 0.1474	Yes	<0.0001
**Base vs. Post2**	0.0635	0.02842 to 0.09851	Yes	<0.0001	0.0568	0.02684 to 0.0868	Yes	<0.0001
**Base vs. Post3**	0.0447	0.009626 to 0.07971	Yes	0.0015	0.0213	-0.008695 to 0.05126	No	0.9221
**Base vs. Post4**	0.0445	0.009456 to 0.07954	Yes	0.0016	0.0145	-0.01547 to 0.04448	No	>0.9999

**Base vs. Post5**	0.0066	-0.02847 to 0.04162	No	>0.9999	-0.0065	-0.03651 to 0.02345	No	>0.9999
**Base vs. Post6**	0.0009	-0.03412 to 0.03597	No	>0.9999	0.0013	-0.02863 to 0.03133	No	>0.9999
**Base vs. Post7**	-0.0063	-0.04133 to 0.02876	No	>0.9999	-0.0074	-0.0374 to 0.02256	No	>0.9999
**Base vs. Post8**	-0.0030	-0.03809 to 0.032	No	>0.9999	0.0009	-0.02906 to 0.0309	No	>0.9999
**Pert7$_L$**								
**Base vs. Pre**	0.0010	-0.02507 to 0.04502	No	>0.9999	0.0090	-0.02093 to 0.03903	No	>0.9999
**Base vs. Post1**	0.1019	0.06684 to 0.1369	Yes	<0.0001	0.1325	0.1026 to 0.1625	Yes	<0.0001
**Base vs. Post2**	0.0860	0.05095 to 0.121	Yes	<0.0001	0.0534	0.02343 to 0.08338	Yes	<0.0001
**Base vs. Post3**	0.0620	0.02698 to 0.09707	Yes	<0.0001	0.0260	-0.003998 to 0.05596	No	0.2113
**Base vs. Post4**	0.0555	0.02044 to 0.09052	Yes	<0.0001	0.0201	-0.009908 to 0.05005	No	>0.9999
**Base vs. Post5**	0.0052	-0.02988 to 0.04021	No	>0.9999	-0.0027	-0.0327 to 0.02725	No	>0.9999
**Base vs. Post6**	0.0020	-0.03309 to 0.037	No	>0.9999	0.0003	-0.02969 to 0.03026	No	>0.9999
**Base vs. Post7**	-0.0019	-0.03699 to 0.0331	No	>0.9999	-0.0098	-0.03982 to 0.02013	No	>0.9999
**Base vs. Post8**	0.0048	-0.03024 to 0.03985	No	>0.9999	-0.0043	-0.03423 to 0.02572	No	>0.9999
**Pert8$_L$**								
**Base vs. Pre**	0.0090	-0.02607 to 0.04402	No	>0.9999	0.0058	-0.02418 to 0.03577	No	>0.9999
**Base vs. Post1**	0.1122	0.07718 to 0.1473	Yes	<0.0001	0.1215	0.09149 to 0.1514	Yes	<0.0001
**Base vs. Post2**	0.0852	0.05014 to 0.1202	Yes	<0.0001	0.0755	0.0455 to 0.1055	Yes	<0.0001
**Base vs. Post3**	0.0641	0.02911 to 0.0992	Yes	<0.0001	0.0450	0.01499 to 0.07495	Yes	<0.0001
**Base vs. Post4**	0.0407	0.005665 to 0.07575	Yes	0.0069	0.0269	-0.003094 to 0.05686	No	0.1549
**Base vs. Post5**	0.0086	-0.02639 to 0.04369	No	>0.9999	0.0046	-0.0254 to 0.03456	No	>0.9999
**Base vs. Post6**	0.0007	-0.03435 to 0.03573	No	>0.9999	0.0026	-0.02737 to 0.03259	No	>0.9999
**Base vs. Post7**	-0.0030	-0.03802 to 0.03207	No	>0.9999	-0.0153	-0.04533 to 0.01463	No	>0.9999
**Base vs. Post8**	0.0053	-0.02974 to 0.04034	No	>0.9999	-0.0019	-0.03185 to 0.0281	No	>0.9999
**Pert9$_L$**								
**Base vs. Pre**	0.0020	-0.03309 to 0.03699	No	>0.9999	-0.0003	-0.03027 to 0.02969	No	>0.9999
**Base vs. Post1**	0.1191	0.08407 to 0.1542	Yes	<0.0001	0.1439	0.1139 to 0.1739	Yes	<0.0001
**Base vs. Post2**	0.0743	0.03927 to 0.1094	Yes	<0.0001	0.0541	0.02413 to 0.08408	Yes	<0.0001
**Base vs. Post3**	0.0540	0.019 to 0.08909	Yes	<0.0001	0.0250	-0.005036 to 0.05492	No	0.2986
**Base vs. Post4**	0.0482	0.01313 to 0.08322	Yes	0.0003	0.0164	-0.01362 to 0.04633	No	>0.9999

**Base vs. Post5**	0.0204	-0.01462 to 0.05546	No	>0.9999	0.0034	-0.02656 to 0.0334	No	>0.9999
**Base vs. Post6**	0.0066	-0.02843 to 0.04165	No	>0.9999	0.0092	-0.02074 to 0.03921	No	>0.9999
**Base vs. Post7**	-0.0008	-0.03587 to 0.03421	No	>0.9999	-0.0030	-0.03298 to 0.02697	No	>0.9999
**Base vs. Post8**	0.0000	-0.03503 to 0.03506	No	>0.9999	0.0005	-0.02945 to 0.03051	No	>0.9999
**Pert10$_R$**								
**Base vs. Pre**	-0.0041	-0.0391 to 0.03099	No	>0.9999	-0.0028	-0.03278 to 0.02718	No	>0.9999
**Base vs. Post1**	0.1018	0.06671 to 0.1368	Yes	<0.0001	0.1116	0.08163 to 0.1416	Yes	<0.0001
**Base vs. Post2**	0.0964	0.06139 to 0.1315	Yes	<0.0001	0.0548	0.02487 to 0.08483	Yes	<0.0001
**Base vs. Post3**	0.0943	0.05931 to 0.1294	Yes	<0.0001	0.0520	0.02203 to 0.08199	Yes	<0.0001
**Base vs. Post4**	0.0883	0.05324 to 0.1233	Yes	<0.0001	0.0514	0.02143 to 0.08139	Yes	<0.0001
**Base vs. Post5**	0.0446	0.009539 to 0.07962	Yes	0.0015	0.0077	-0.02229 to 0.03767	No	>0.9999
**Base vs. Post6**	0.0109	-0.02412 to 0.04597	No	>0.9999	0.0056	-0.02441 to 0.03555	No	>0.9999
**Base vs. Post7**	0.0007	-0.0343 to 0.03579	No	>0.9999	-0.0003	-0.03029 to 0.02967	No	>0.9999
**Base vs. Post8**	-0.0087	-0.04379 to 0.0263	No	>0.9999	0.0011	-0.0289 to 0.03106	No	>0.9999
