# Supplementary material for: Retention, savings and interlimb transfer of reactive gait adaptations in humans following unexpected perturbations
Source: Commun Biol. 2018 Dec 14;1:230. doi: 10.1038/s42003-018-0238-9 (PMC6294781; doi:10.1038/s42003-018-0238-9)
Supplement: Supplementary file 3 — Description of Additional Supplementary Files [file 42003_2018_238_MOESM3_ESM.docx]

**Description of Additional Supplementary Files**

**File Name**: Supplementary Data 1

**Description**: This excel file includes the data used to produce each of the results figures included in the article.
